# “Surgically Treated COVID-19-Positive Trauma Patients Had a Higher Fatality Rate” - A Rural District General Hospital’s Perspective in the United Kingdom

**DOI:** 10.7759/cureus.18905

**Published:** 2021-10-19

**Authors:** Soubhik Ghosh, Anoop John, Prashanth D'sa, Bijai Kurian, Peace Ayodele, Anirudh Gadgil

**Affiliations:** 1 Trauma and Orthopaedics, Glangwili General Hospital, Carmarthen, GBR; 2 Trauma and Orthopaedics, University Hospital of Wales, Cardiff, GBR

**Keywords:** fracture, trauma, mortality, pandemic, covid-19

## Abstract

Introduction

Our study analyses the influence of the COVID-19 pandemic on perioperative death in elderly patients undergoing surgery for fractures who test positive for the virus during their admission in a rural hospital setting in the UK.

Methods

One hundred and fifty-six consecutive patients with age more than 75 years, who underwent surgery for fractures in Glangwili General Hospital during the second wave of the pandemic between 20th November 2020 and 20th January 2021, were included in this study. The 28-day mortality rate was estimated, and the results were compared to a matched cohort of patients from a similar duration before the pandemic (20th November 2019 to 20th January 2020).

Results

A total of 41 out of 156 patients were tested positive for COVID-19 in this study cohort. The overall 28-day mortality rate was 8.9% (n=15 patients) in comparison to 4% (n=8) in the comparative cohort of 196 patients from the pre-pandemic era. Of the 41 patients who tested positive for COVID-19, 11 patients died within 28 days of surgery, resulting in a mortality rate of 26.8% with a relative risk of 7.7(p=0.0461). Furthermore, 91% (n=10) of COVID-19-positive patients who died had an underlying cardiac disease and/or proximal femoral fractures. The 28-day mortality rate in those tested negative for COVID-19 was 3.5% (n=4).

Conclusion

There is a significantly increased risk of death in the perioperative period on contracting COVID-19, in patients who are 75 years of age or older, especially those with associated cardiac comorbidities and who have sustained proximal femoral fractures.

## Introduction

During the height of the first wave of the COVID-19 pandemic, most elective surgeries in the United Kingdom were halted or significantly reduced in the anticipation that contracting COVID-19 infection perioperatively may increase the risk of complications and death [1]. This also allowed for combining healthcare resources and redeployment of healthcare staff to prioritize medical emergencies and COVID-19 treatment [2,3]. Fractures and soft tissue injuries were managed conservatively when feasible, to avoid surgery and hospital admissions during the COVID-19 surge [4]. However, patients with some long bone fractures and elderly patients with proximal femoral fractures had limited choice but to undergo surgery due to the certainty of a poor outcome with conservative management. Recently published studies have demonstrated that COVID-19 pandemic has resulted in increased mortality rates in hip fracture patients who undergo surgery [5-8]. We carried out this retrospective observational cohort study to quantify the influence of COVID-19 on perioperative death in trauma patients who underwent surgery in a rural hospital, so we could counsel the patients and their family regarding the risks of surgery during the pandemic. In our hospital that caters to a large rural area of west Wales, COVID-19 infections during the first wave of the pandemic were very low. However, during the second wave in the months of December 2020 and January 2021, we had a full-blown COVID-19 infection upsurge in the community, and hence the chosen time period to observe the influence of the effect of COVID-19 on perioperative mortality in trauma patients undergoing surgery.

We hypothesized that there is an increased risk of mortality in patients who tested positive for COVID-19 to those who tested negative and increased overall mortality rate during the pandemic period compared to the pre-pandemic era in surgically treated fractures in those aged 75 or more.

## Materials and methods

All consecutive patients of age 75 or more, who underwent trauma surgery between the time period of 20th November 2020 and 20th January 2021 in our hospital, were included. Data on patients who tested positive for COVID-19 since admission and 28-day mortality were obtained using the hospital electronic database Welsh Patient Administration System. We obtained data regarding age, sex , comorbid condition like pre-existing cardiac disease, diabetes mellitus, hypertension, clotting disorder, pre-injury mobility and functional status, level of independence, social status, date of admission, date of operation, postoperative mobilization, date of diagnosis of COVID-19 infection, duration since diagnosis, and death due to COVID-19 infection. A matched cohort of patients admitted in the pre-pandemic era who underwent surgery for trauma between 20th November 2019 and 20th January 2020 was chosen as a comparative group. We then obtained similar data from this group of patients for comparison. This study was approved by the hospital department as a service evaluation. We also compared the mortality rates between those who were COVID positive and COVID negative to estimate the effects of the pandemic on perioperative mortality in our cohort of trauma patients.

Statistical analysis

Two-tailed Student's t-tests were used to evaluate differences in continuous variables while Fisher's exact test or chi-square test was used to evaluate differences in categorical data. Fisher's exact test was used in proportion comparisons where values in any cells fell below 5. The chi-square test was used in proportion comparisons where values in all cells were above 5. Significance was defined as p ≤0.05. Data were analyzed using MedCalc Statistical Software version 19.8 (MedCalc Software Ltd, Ostend, Belgium). We do confirm that human rights were not violated in this study and our study did not involve any animal participation.

## Results

The study cohort consisted of 156 consecutive patients who underwent surgical management for lower limb fractures. All patients were operated on within an average of two days of admission and mobilized as soon as it was safe under the supervision of physiotherapists. Of these, 15 patients died postoperatively, resulting in an overall 28-day mortality rate of 9.61%. A total of 41 patients (26.3%) in the study cohort tested positive for COVID-19 during admission, of which 11 patients died within 28 days of surgery (mortality rate=26.8%). The mean age of COVID-19-positive patients who died was 86.1 years (range 75-92 years) with an equal gender distribution (male:female ratio 1:1). The average time since diagnosis of infection to death was 12.5 days. Amongst the patients who tested positive for COVID-19 and died, nine had pre-existing cardiac disease, five patients were diabetic, five were hypertensive, three had a previous history of deep vein thrombosis, six patients did not have very good pre-injury mobility and were not functionally independent, and rest of the five patients were mobile with the help of a walking frame and were reasonably independent. Seven patients lived in care homes and the rest lived with their family at home. Ten had sustained proximal femoral fractures and underwent either osteosynthesis or cemented hemiarthroplasty. 73.3% of patients who died in the study cohort had tested positive for COVID-19 during their in-patient stay. Of the remaining 115 patients who tested negative for COVID-19, the 28-day mortality rate was 3.5% (n=4). The difference in mortality rates between those who tested positive to those who tested negative for COVID-19 was statistically significant (relative risk [RR]=7.7, 95% CI: 2.60-22.88, p=0.0002). Table 1 shows comparative mortality figures between COVID-positive and -negative groups.

**Table 1:** Comparison of mortality rates between COVID-positive and COVID-negative groups of patients.

Total number of patients n= 156	Number of patients who died within 28 days postoperative period	Mortality rate in %
COVID positive = 41	11	26.8
COVID negative = 115	04	3.5

A total of 194 patients underwent surgery between 20th November 2019 and 20th January 2020. Their mean age was 85.3 years (range 76-90) with a male-to-female distribution of 1.2:1. Of these, eight patients died within 28 days following surgical procedure with a mortality rate of 4% (Table 2). There was a 2.3 times increase (RR) in mortality in trauma patients operated during the COVID-19 pandemic compared to the pre-COVID era, and this difference was statistically significant (95% CI: 1.01-5.35, p=0.0461).

**Table 2:** A comparative analysis of mortality figures between COVID pandemic and pre-pandemic time periods.

Type	COVID time period (20thNovember, 2020 to 20thJanuary, 2021)	Pre-COVID time period (20thNovember, 2019 to 20thJanuary, 2020)
Male:female	1:1	1.2:1
Total number of patients operated	156	194
Total number of patients died within 28 days of operation	15	8
Mortality rate	9.61%	4%

## Discussion

Our retrospective comparative review has found that there is more than seven-fold increased risk of death in the perioperative period on contracting COVID-19, in patients who are 75 years of age or older in a rural hospital setting. Besides, there is a statistically increased overall mortality rate in patients being operated for fractures during the pandemic compared to the pre-pandemic era, thus confirming our hypothesis.

A large multi-centre international cohort study found an overall 30-day mortality rate of 23.8% amongst 1,128 COVID-19-positive patients undergoing surgery [1]. The mortality rate was found to be higher in those undergoing emergency surgery (214/824, 26.0%) and orthopaedic trauma surgery (86/299, 28.8%). Our study shows similar mortality rates of 26.8% amongst the COVID-19-positive patients who underwent trauma surgery. A meta-analysis by Lim and Pranata showed that COVID-19 infection was associated with a seven-fold increase in risk (RR 7.45 [95% CI: 2.72-20.43], p<0.001; I^2^: 68.6%) of mortality in hip fracture patients. The analysis included 984 participants with a pooled prevalence of COVID-19 of 9%, with mortality rate amongst COVID-19-positive patients undergoing hip fracture surgery being 28% compared to 10.3% in COVID-19-negative patients. Another systematic review and meta-analysis by Wang et al. analyzing the risk of early mortality in hip fracture patients with COVID-19 infection who have undergone surgical intervention included 16 studies and found the relative risk for postoperative mortality in COVID-19-positive patients compared to COVID-negative patients to be 5.66 (95% CI: 4.01-7.98; p<0.001). Results from both meta-analyses are similar to our study; however, our study cohort includes all surgically treated fractures in the elderly rather than only hip fractures.

Strengths of this study include (age and sex) matched comparative cohort, whereas the weaknesses include a single-centre data with a small sample size, and its retrospective design. Retrospective studies have an inherent selection bias and do not include potential confounding variables. The generalizability of findings of our study to other emergency and elective surgical procedures, and other geographical settings remains unknown. A large nationwide multi-centre study should allow for more appropriate quantification of effects of this pandemic in surgically treated trauma patients, may improve prognostic stratification, and identify additional risk factors for increased mortality at different hospital settings.

To the best of our knowledge, this is the first study from UK reporting a rural hospital’s perspective of effect of COVID-19 on mortality rates in surgically treated trauma patients. Our study shows significantly increased risk of mortality amongst surgically treated COVID-19-positive trauma patients compared to those who tested negative for COVID-19.

## Conclusions

There is more than seven-fold increased risk of death in the perioperative period on contracting COVID-19, in patients who are 75 years of age or older, especially those with associated cardiac comorbidities and who have sustained proximal femoral fractures. This finding should allow us to appropriately inform the patients and their family during admission for surgical procedures in trauma in a rural hospital setting.
